# The Unrecognized Chancre

**Published:** 1902-03

**Authors:** 


### Abstracts and Selections.

### The Unrecognized Chancre.

In the *International Medical Magazine* for October William S. Gottheil calls attention to the frequent insignificance and fugacity of the syphilitic initial lesion, which leads to its non-recognition in quite a large proportion of cases. Ignorance of its occurrence, and not voluntary falsification, is the cause of the frequent absence of a syphilitic history in undoubtedly specific cases. The author calls attention to the following points of diagnosis:

1. The presence of a tumor as the original lesion. In its essence, and invariably at the beginning, the chancre is a small round cell accumulation in the skin or subcutaneous tissue. Ulceration may occur, and usually does, or even phagadaenism; but these are accidental, and epiphenomena and almost invariably the specific induration is appreciable at the base of the lesion.

2. The tumor is indolent, painful and recalcitrant to treatment.

3. A peculiar and characteristic "stony" induration of the nearest lymphatic glands accompanies it, different from the general adenopathy that occurs later as a consequence of the systemic infection. Other lesions, as gummata, do not show it.

4. Chancre runs its full course in a few weeks, whilst tuberculosis takes months, and carcinoma even years, for its development.

5. The well known signs of general luetic infection, osteoconic pain, cephalalgia, synovitis, general lymphadenitis, exanthem, etc., must be carefully and persistently searched for in every suspicious case. They may be so slight as to entirely escape careless examination.

